# 

**Published:** 1843-02-04

**Authors:** 


CONTENTS.

A Course of Clinical Lectures, delivered at the Hôtel-Dieu, Paris, for the Session 1842–'43. By A. F. Chomel. Lecture 1.
Case 1. Chronic meningitis, with slight hemiplegia—symptoms of compression—death—considerable serous effusion in the ventricles—anatomical characters of meningitis, . . 13
Case 2. Partial hemiplegia of left side—Paralysis of the right superior lid, and of the motor muscles of the eye—The consequence of repeated epileptic attacks, . . . . . . 14
Case of Epilepsy, induced by a blow upon the head, successfully treated. By Isaac Parrish, M. D. . . . . . 14
Note to Dr. Horner's case of luxation of the cervical vertebræ, . . . . 17
*University of Pennsylvania.* Clinic of Dr. W. Poyntell Johnston, concluded.
Case 4. Lacrymal tumour—operation—occlusion of the nasal duct—obliteration of the lower portion of the lacrymal sac—great epiphora—perforation of the os unguis—cure, . . 17
Case 5. Syphilis three years since—great exposure during a whaling voyage—caries of the upper maxilla, 18
Case 6. Extraction of second large molar tooth of lower jaw followed by great swelling, and necrosis of the alveolar processes—abscess near the chin—through the opening, the ramus of the jaw can be felt denuded, . 19
*Jefferson Medical College.*
Clinic of Professor Mütter.
Case. Ulcer of the lid of seven years standing--cause unknown—blepharoplastic operation for its relief—results of operation, . . . 19
*Philadelphia Hospital.* Professor Pancoast's Clinic, concluded.
Case 4. Chronic enlargement of the tonsil gland of each side, . . . 21
Case 5. Fracture of the clavicle, . 22
Case 6. Frost-bite of both hands, . 22
Case 7. Operation for hydrocele, . 22
Case 8. Amputation of the toe at the metatarso-phalangeal joint, . . 22
*Wills' Hospital.* Clinic of Dr. Isaac Parrish,
Case 1. Encysted tumour of the upper eyelid, . . . . . 22
Case 2. Ophthalmia, ulcers, and opacity of the cornea, . . . 23
*Bibliographical Notice.*
A system of Anatomy, for the use of Students of Medicine. By Caspar Wistar, M. D. With notes and additions. By W. E. Horner, M. D. Entirely remodelled, and illustrated with more than two hundred engravings. By J. Pancoast, M. D. . 23
*Foreign Correspondence,* . . . 24

